# Extracting the electronic structure signal from X-ray and electron scattering in the gas phase

**DOI:** 10.1107/S1600577524000067

**Published:** 2024-02-22

**Authors:** Thomas Northey, Adam Kirrander, Peter M. Weber

**Affiliations:** a Department of Chemistry, Brown University, Providence, RI 02912, USA; b Physical and Theoretical Chemistry Laboratory, Department of Chemistry, University of Oxford, South Parks Road, Oxford OX1 3QZ, United Kingdom; ESRF – The European Synchrotron, France

**Keywords:** X-ray scattering, electron scattering, gas phase, *ab initio*

## Abstract

X-ray and electron scattering from free gas-phase molecules is examined using the independent atom model (IAM) and *ab initio* electronic structure calculations. The IAM describes the effect of the molecular geometry on the scattering, but does not account for the redistribution of valence electrons due to, for instance, chemical bonding. By examining the total, *i.e.* energy-integrated, scattering from three molecules, fluoroform (CHF_3_), 1,3-cyclohexadiene (C_6_H_8_) and naphthalene (C_10_H_8_), the effect of electron redistribution is found to predominantly reside at small-to-medium values of the momentum transfer (*q* ≤ 8 Å^−1^) in the scattering signal, with a maximum percent difference contribution at 2 ≤ *q* ≤ 3 Å^−1^. A procedure to determine the molecular geometry from the large-*q* scattering is demonstrated, making it possible to more clearly identify the deviation of the scattering from the IAM approximation at small and intermediate *q* and to provide a measure of the effect of valence electronic structure on the scattering signal.

## Abstract 2

A new method to determine molecular structure from large-angle scattering for X-ray and electron scattering from free gas-phase molecules is described.

## 1. Introduction

### 1.1. Background on gas-phase X-ray and electron scattering

Scattering has provided an indispensable tool in advancing our understanding of the structure of matter (von Laue, 1915 ▸; Bragg & Bragg, 1915 ▸; Watson & Crick, 1953 ▸; Perutz *et al.*, 1960 ▸). Gas-phase scattering from molecules was a key component in early advances (Debye, 1915 ▸; Debye *et al.*, 1929 ▸; Debye, 1930 ▸; Pirenne, 1939 ▸, 1946 ▸) and the invention of X-ray free-electron lasers (XFELs) has sparked a renewed interest in gas-phase X-ray scattering (Küpper *et al.*, 2014 ▸; Minitti *et al.*, 2014 ▸), not the least in the domain of ultrafast X-ray scattering (Minitti *et al.*, 2015 ▸; Glownia *et al.*, 2016 ▸; Kirrander *et al.*, 2016 ▸; Stankus *et al.*, 2019 ▸; Ruddock *et al.*, 2019 ▸; Yong *et al.*, 2020 ▸, 2021*a* ▸; Gabalski *et al.*, 2022 ▸; Odate *et al.*, 2023 ▸), alongside advances in ultrafast electron diffraction (UED) experimental capabilities (King *et al.*, 2005 ▸; Sciaini & Miller, 2011 ▸; Weathersby *et al.*, 2015 ▸; Zandi *et al.*, 2017 ▸). We note that in the context of scattering, X-ray and electron scattering are close analogues (Stefanou *et al.*, 2017 ▸; Ma *et al.*, 2020 ▸) and in this paper we consider both.

A chemical bonding effect has been observed in the electron scattering signal of molecules in the gas phase, observed mainly at small scattering angles (Iijima *et al.*, 1965 ▸; Fink & Kessler, 1967 ▸; Duguet & Jaegle, 1975 ▸; Hirota *et al.*, 1981 ▸; An-Ding & Xiao-Lei, 1995 ▸). This has been theoretically studied to quantify the effect in a number of molecules (Bonham & Iijima, 1963 ▸; Wang *et al.*, 1994 ▸; Hoffmeyer *et al.*, 1998 ▸; Shibata *et al.*, 1999 ▸, 2002 ▸). We discuss this effect in the total X-ray and electron scattering of gas-phase molecules, further quantifying the error that occurs in the structure determination, and proposing an approach to separate out the molecular structure contribution in the signal from the contribution from the electronic structure.

### 1.2. Aim of the paper

The aim of this paper is to establish a procedure to identify the valence electronic structure contribution to the molecular scattering signal in the gas-phase and to determine the molecular structure without resorting to full *ab initio* calculations of the scattering signal. The valence contribution is characteristic of the redistribution of electrons away from spherical atom-centred distributions, predominantly due to electrons in the valence shells forming molecular orbitals as in chemical bonding and it may be comparatively localized in *q* (Bredtmann *et al.*, 2014 ▸). Although scattering is commonly viewed as a method to probe molecular geometry, X-rays scatter from all the electrons in the target and thus the scattering relates to the electron density (Ben-Nun *et al.*, 1996 ▸; Kirrander, 2012 ▸; Suominen & Kirrander, 2014 ▸; Northey *et al.*, 2014 ▸, 2016 ▸; Northey & Kirrander, 2019 ▸), and even the pairwise correlation between electrons (Moreno Carrascosa *et al.*, 2019 ▸, 2022 ▸; Zotev *et al.*, 2020 ▸). As a consequence, effects such as the redistribution of electrons due to chemical bonding, the delocalization of electrons in aromatic rings, and the localization of electrons in valence molecular orbitals appear in the scattering signal. Time-resolved experiments can thus detect the rearrangement of electrons due to photoexcitation (Yong *et al.*, 2020 ▸), changes in inelastic scattering due to changes in electronic state populations (Yang *et al.*, 2020 ▸), dynamic charge transfer (Yong *et al.*, 2021*b* ▸), the breaking of chemical bonds (Ruddock *et al.*, 2019 ▸), or carry out tomography on rotational wave packets (Zhang *et al.*, 2021 ▸). Interesting future directions for scattering experiments are also actively explored via simulations (Mu *et al.*, 2023 ▸; Bertram *et al.*, 2023 ▸). Given sufficient time-resolution, it should become possible to track the dynamics of electrons in a molecular system (Simmermacher *et al.*, 2019*a* ▸,*b* ▸; Ziems *et al.*, 2023 ▸). These opportunities apply to both X-ray and electron scattering, however with additional terms in the electron scattering due to the interactions between the incoming electrons and the nuclei (see Section 2).

For gas-phase scattering, the absence of a crystalline lattice means that energy-integrating detectors pick up the total, rather than just the elastic scattering. In this paper, we use the independent atom model (IAM) approximation (Debye *et al.*, 1929 ▸; Debye, 1930 ▸) of the scattering signal as a baseline that does not include any redistribution of electrons due to bonding, as the IAM assumes a non-interacting spherical distribution of electrons around each individual atom. These results are compared with accurate *ab initio* calculations of the total X-ray scattering that fully account for the redistribution of electrons (Moreno Carrascosa *et al.*, 2019 ▸; Zotev *et al.*, 2020 ▸; Carrascosa *et al.*, 2022 ▸). The difference between the IAM and the *ab initio* signal is identified as the valence electronic structure component. However, it is important to note that the exact molecular geometry is not necessarily known *a priori*. We therefore require a procedure to determine the molecular geometry as accurately as possible before the electronic component can be calculated. We show in this paper that the molecular geometry can be reliably determined from the large values of the momentum transfer *q*, while small and intermediate *q* values are most affected by electronic effects. In doing this, we use a recently developed simulated annealing algorithm to fit the molecular geometry to a target X-ray signal (Northey *et al.*, 2024 ▸) for various ranges of the momentum transfer vector *q*.

## 2. Methods

### 2.1. X-ray and electron scattering

#### 2.1.1. *Ab initio* calculation

The *ab initio* X-ray and electron scattering calculations were carried out using an in-house code from the Kirrander group (Northey *et al.*, 2014 ▸; Moreno Carrascosa *et al.*, 2019 ▸; Zotev *et al.*, 2020 ▸) that calculates the scattering signal directly from the molecular wavefunction expressed in a Gaussian basis and obtained via *ab initio* electronic structure methods, such as Hartree–Fock (HF) or multiconfigurational wavefunction methods (CASSCF, MRCI, MCCI *etc*). The code calculates the total scattering, *i.e.* both the elastic and inelastic components of the signal. In this paper, HF theory with the 6-31G* Pople basis set is used.

#### 2.1.2. Independent atom model

According to the IAM, for X-ray scattering from an isotropic ensemble of *N*_at_-atom molecules the total scattering intensity is

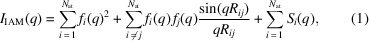

where the first sum constitutes the atomic scattering, *I*_at_(*q*), and runs over all atoms *i* with tabulated atomic X-ray scattering factors *f*_*i*_(*q*) (Prince, 2006 ▸). The first sum contains no structural information about the molecule; instead, structure is contained in the second molecular scattering term, *I*_mol_(*q*), a double sum which runs over all pairs of atoms *i* and *j* (excluding *i* = *j*). This term involves the distance between atoms, *R*_*ij*_ = |**R**_*i*_ − **R**_*j*_|, where **R**_*i*_ and **R**_*j*_ are the positions of atoms *i* and *j*, respectively. The final term accounts for inelastic scattering and is a sum of tabulated inelastic scattering factors, *S*_*i*_(*q*). The amplitude of the scattering vector is *q* = |**q**|, defined as **q** = **k**_1_ − **k**_0_, with **k**_1_ and **k**_0_ the wavevectors of the scattered and incident X-ray photons. Finally, we note that equation (1) is appropriate for scattering from rotationally averaged free gas-phase molecules in their electronic ground state, as considered in this paper.

For electron scattering, the Mott–Bethe formula (Mott, 1930 ▸) can be used to transform the X-ray atomic factors to electron factors, 

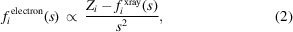

with proportionality constant 2*m*_e_ *e* ^2^/ℏ. This means that the IAM electron scattering is very similar to the X-ray scattering equation aside from the 1/*s* ^2^ and *Z*_*i*_ terms, where *Z*_*i*_ is the atomic number of atom *i*, due to the additional scattering of the electrons by the positive charge of the nuclei. By convention, in electron scattering the scattering vector is denoted as *s* instead of *q*.

In this paper, comparison between *ab initio* and IAM scattering is in terms of the percent difference, defined as 

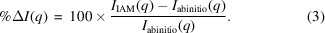

Note that this is a percentage, and that the reference signal *I*_abinitio_(*q*) is subtracted from *I*_IAM_(*q*) and also appears in the denominator.

### 2.2. Fitting procedure

A recently developed simulated annealing (SA) approach is used to fit the predicted IAM signal to the target data. This approach is described in detail by Northey *et al.* (2024 ▸). It minimizes the target function, 

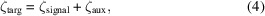

by changing the molecular geometry **R**′ and recalculating ζ_targ_ iteratively, where 

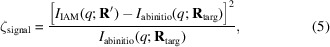

for the predicted X-ray (or electron) IAM scattering signal which depends on **R**′, and the target signal is calculated using the *ab initio* scattering code, where **R**_targ_ is the target geometry. Auxiliary harmonic oscillator terms ζ_aux_ = 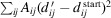 are included in ζ_targ_, with interatomic distances 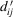 and 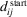 between atoms *i* and atom *j* corresponding to distances from **R**′ and the starting geometry **R**_start_, respectively. The index *i* = 1, 2,…, *N*_at_ runs over each atom in the molecule, whereas index *j* ≠ *i* runs over each nearest-neighbour and second-nearest-neighbour atom (counting from atom *i*). This restrains the molecular geometry, ensuring that the simulated annealing algorithm does not waste effort exploring unphysical regions of the conformational space. The auxiliary terms have weighting factors *A*_*ij*_ which are tuned such that the scattering term ζ_signal_ is the predominant driving force in the minimization, *i.e.* ζ_signal_/ζ_aux_ ≃ 10.

The fitting procedure minimizes the squared-difference functions contained in equation (4) iteratively by randomly perturbing the molecular geometry along normal modes and accepting perturbations if the fit improves. The method is robust and can escape local minima by accepting non-favourable (uphill) steps with non-zero probability, corresponding to an effective temperature. Simulated annealing works quite well here, but other optimization methods should be capable of reproducing the same results. Notably, the focus of the current paper is not the overall optimal structure determination but rather to establish the information content in various *q*-ranges of the scattering signal. Additional data, for instance from other experiments or *ab initio* calculations, which are exploited in structural retrieval methods such as the SARACEN method developed by Mitzel & Rankin (2003 ▸), would be counterproductive in this context as they would distort this analysis. In this work, the target geometry is the Hartree-Fock(HF)/6-31G* ground state optimized geometry, **R**_targ_ = **R**_0_, calculated using *MOLPRO* (Werner *et al.*, 2012 ▸). Notably, in an experiment, the target geometry would not be known *a priori*; the goal is to find it by sampling around a reasonable initial guess.

The starting geometry for the fitting procedure is initialized by small random perturbations along each mode, away from **R**_0_. Thus, the starting geometry is close to the target geometry, **R**_start_ ≃ **R**_targ_, and the fitting procedure predominantly depends on the difference between the IAM and *ab initio* signals rather than an extensive conformational search. A frequency calculation on the ground state **R**_0_ geometry is performed to obtain the normal mode unit vectors, which are used to sample all dimensions of the nuclear coordinate space when minimizing the target function. The final molecular geometry **R**_best_ is determined from the minimum of ζ_targ_. Due to the stochastic nature of the SA algorithm, it was run 20 times for each fitting and the outcome with the lowest ζ_targ_ is selected. This avoids getting stuck in higher-lying minima and increases the probability that a structure close to the global minimum is obtained.

**Figure 1:**
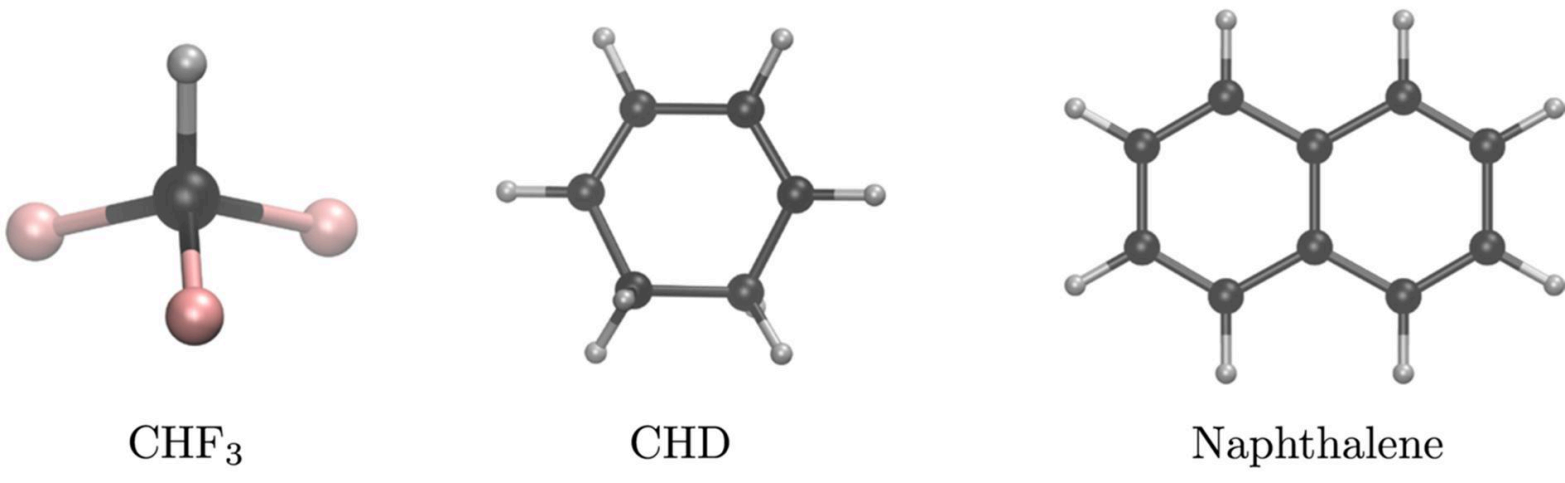
The three molecules fluoroform (CHF_3_), 1,3-cyclohexadiene (C_6_H_8_, CHD) and naphthalene (C_10_H_8_).

A metric used in this work to assess how close a given molecular geometry is to the reference geometry is the mean absolute percent deviation (MAPD) (Yong *et al.*, 2021*c* ▸), defined as 

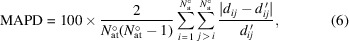

for the total number of atoms of interest 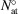 , where 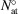 ≤ *N*_at_. In this work, the non-hydrogen atoms are chosen in the calculation of the MAPD, *i.e.* 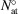 equals the number of non-hydrogen atoms. The distances between atom *i* and atom *j* are *d*_*ij*_ and 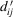 , where the prime (′) denotes the reference structure, which is the ground state optimized structure **R**_0_ unless otherwise specified. In the following, we proceed to consider three molecules: fluoroform (CHF_3_), 1,3-cyclohexadiene (C_6_H_14_, CHD) and naphthalene (C_10_H_8_), which are shown in Fig. 1 ▸.

## 3. Results

### 3.1. IAM and *ab initio* scattering calculations

**Figure 2:**
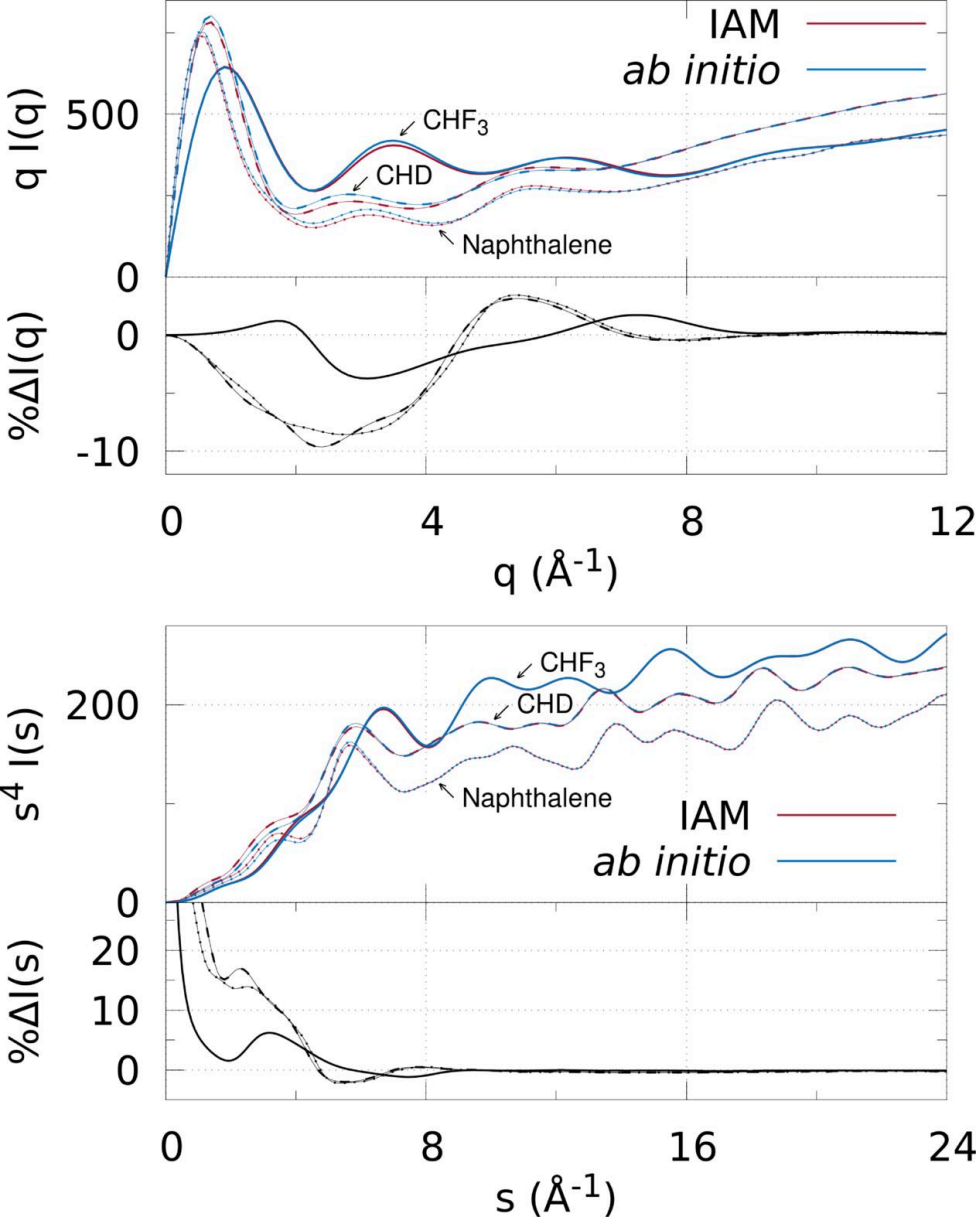
Comparison between IAM and *ab initio* scattering (top figure: X-ray scattering; bottom figure: electron scattering) for the three molecules CHF_3_, CHD and naphthalene in their optimized ground-state geometries **R**_0_. The scattering signals are shown in the top panels (IAM by red lines and *ab initio* by blue), with the signals scaled by *q* and *s* ^4^, respectively, *i.e.* *qI*(*q*) and *s* ^4^ *I*(*s*). In both top and bottom figures the naphthalene signal has been multiplied by 0.5 for visualization purposes. The bottom panels show percent differences between *ab initio* and IAM scattering, as defined in equation (3). Note that the electron scattering 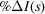 becomes very large at small *s* due to division by small numbers, so the *y*-axis is truncated.

The top panels in Fig. 2 ▸ show a comparison between IAM and *ab initio* X-ray (top) and electron (bottom) scattering for the three molecules in Fig. 1 ▸. The *I*(*q*) signal is multiplied by *q* to enhance the visibility of the signal at large *q* [see the unscaled scattering intensity *I*(*q*) plotted in Fig. S1 of the supporting information]. The *I*(*s*) signal is multiplied by *s* ^4^ for the same reason. Often in electron scattering experiments, *M*(*s*) = *I*_mol_(*s*)/*I*_at_(*s*) is plotted, which cancels out the *s* ^−4^ Rutherford scattering term in *I*(*s*); however, the *ab initio* *I*(*s*) signal in this work cannot be decomposed into separate *I*_mol_(*s*) and *I*_at_(*s*) terms, so *s* ^4^ *I*(*s*) is shown instead, cancelling the Rutherford scaling. The bottom panels in Fig. 2 ▸ show the percent difference [as in equation (3)] between IAM and *ab initio* scattering.

For X-ray scattering, all three molecules are similar in that the IAM underestimates signal in the approximate range 0 ≤ *q* ≤ 4.6 Å^−1^ (0 ≤ *q* ≤ 5.9 Å^−1^ for CHF_3_), albeit in CHF_3_ IAM slightly overestimates for *q* < 2.1 Å^−1^, and in all three IAM overestimates the scattering at larger *q* (approximately 4.6 ≤ *q* ≤ 8.4 Å^−1^ for CHD and naphthalene, and approximately 5.9 ≤ *q* ≤ 8.8 Å^−1^ for CHF_3_) up until *q* ≳ 8 Å^−1^ when IAM and *ab initio* become very similar; excellent agreement is seen here with 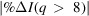 < 0.3%. The peak percent differences for CHD and naphthalene are relatively large, with 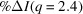 = −9.6% and 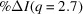 = −8.6%, respectively. They are pure hydrocarbons which have been reported to contain larger chemical bonding effects compared with molecules such as CCl_4_, N_2_, O_2_ and CS_2_ (Shibata *et al.*, 2002 ▸). This is due to delocalization of electrons by chemical bonding, which the IAM does not take into account, including double and triple bonds, aromatic rings, and hydrogen atoms bonded to heavier atoms. Conversely, CHF_3_ has a smaller peak percentage difference of 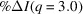 = −3.7% due to the three polarized C–F single bonds and an absence of double bonds or delocalized electrons, which means that the molecule is quite well described by IAM.

Similar to X-ray scattering, the electron scattering results show a substantial difference between IAM and *ab initio*, with the IAM performing the best for CHF_3_, whereas CHD and naphthalene have larger peaks in percentage difference 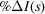 . The maximum peak differences for each molecule are: CHF_3_ has 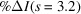 = 6.2%, CHD has 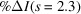 = 17.0%, and naphthalene has 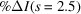 = 13.9%. These peak percent difference values are similar to the X-ray scattering result in terms of magnitude and molecule order, showing that the redistribution of electrons away from atom-centred spherical distributions is similarly visible. Finally, at 8 < *s* < 24 Å^−1^ the mean absolute percent difference is 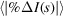 < 0.3% for each molecule, comparable with the X-ray scattering results at 8 < *q* < 12 Å^−1^.

### 3.2. Fitting to the target signal

**Figure 3:**
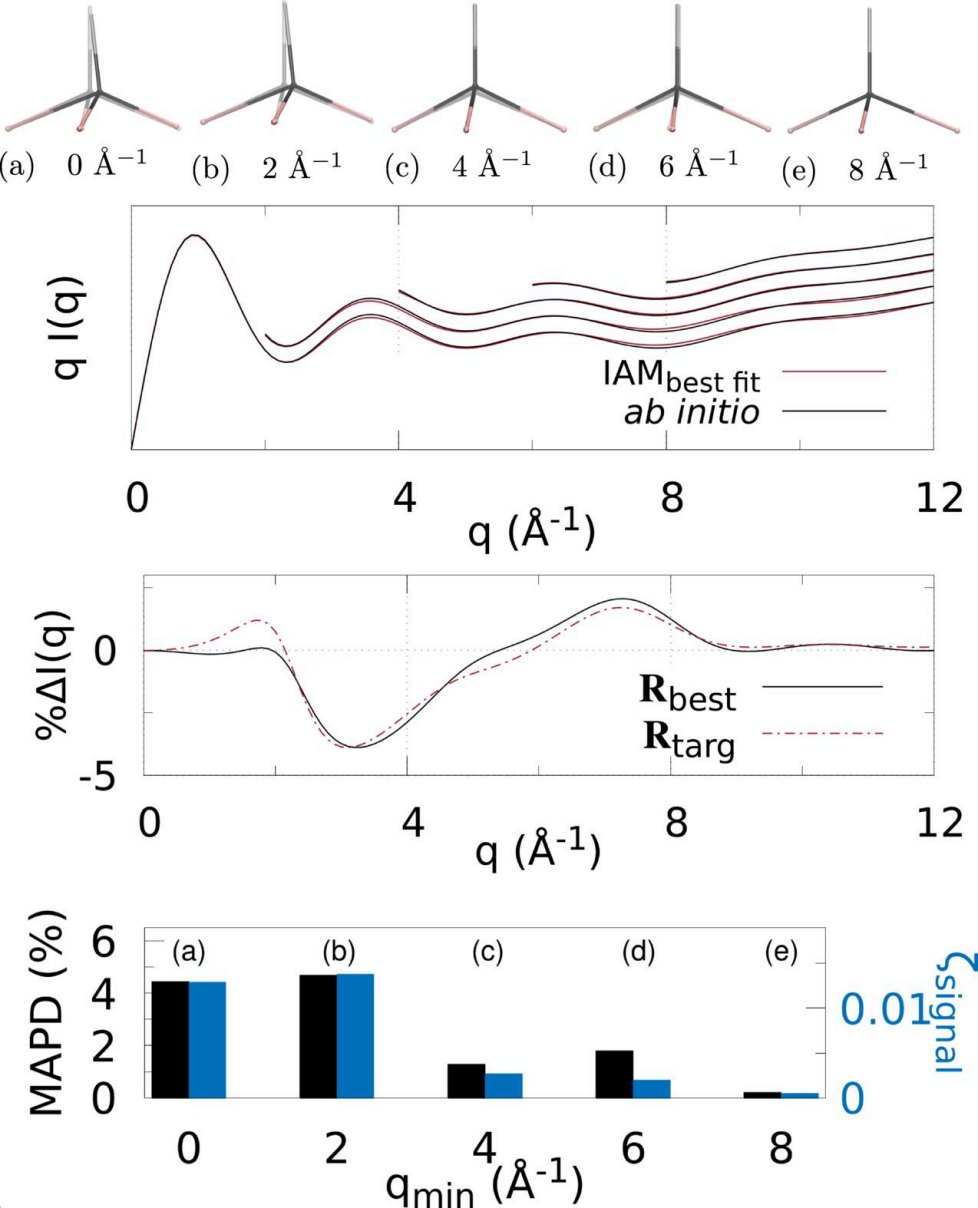
X-ray scattering for CHF_3_ using different *q*-ranges for structure determination: (*a*)–(*e*) The best fit geometry for each *q*_min_ (solid) versus the target structure **R**_targ_ (translucent), where *q* ∈ [*q*_min_, *q*_max_], and *q*_min_ = [0, 2, 4, 6, 8] Å^−1^ and *q*_max_ = 12 Å^−1^. (Top) The IAM best fits to the *ab initio* X-ray scattering calculated at **R**_targ_ as a function of *q*-range. The *q*_min_ ≥ 2 Å^−1^ curves are shifted vertically for visibility. (Middle) The percent difference 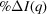 [equation (3)] (solid black line) for the lowest ζ_targ_ structure. For comparison, 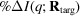 is also shown, using *I*_IAM_(*q*; **R**_targ_) and *I*_abinitio_(*q*; **R**_targ_). (Bottom) Bar chart showing the MAPD [equation (6)] and ζ_signal_ for each value of *q*_min_.

Figs. 3 ▸ ▸–5 ▸ and 6 ▸ ▸–8 ▸ show the results of fitting the IAM signals to *ab initio* X-ray and electron scattering signals, respectively. The predicted data are *I*_IAM_(*q*) for the X-ray scattering, as in equation (1) [not *qI*(*q*) as shown in the figures], and *s* ^4^ *I*_IAM_(*s*) for electron scattering. The corresponding target data is calculated by *ab initio* scattering theory at **R**_targ_ = **R**_0_ for both X-ray and electron scattering, *i.e.* *I*_abinitio_(*q*) or *s* ^4^ *I*_abinitio_(*s*), respectively.

Various *q* (or *s*) ranges were fitted to assess the accuracy of IAM as a function of the values of the momentum transfer, to find the region most suitable to determine the molecular geometry with minimal non-IAM contamination. This, correspondingly, is the region where the valence electronic structure component of the signal is strongest (that is, where the IAM and *ab initio* signals are significantly different).

At the top of each figure the best-fit molecular geometries **R**_best_ (in solid) are shown together with the reference geometries **R**_targ_ (translucent) for the various *q* (or *s*) ranges. For X-ray scattering, the top graph shows the best-fit signal scaled by *q*, *qI*_IAM_(*q*; **R**_best_), compared with *qI*_abinitio_(*q*; **R**_targ_) for each signal range *q*_min_ ≤ *q* ≤ *q*_max_. For electron scattering, similarly the best fits for each range are shown *s* ^4^ *I*_IAM_(*s*; **R**_best_) compared with *s* ^4^ *I*_abinitio_(*s*; **R**_targ_) for *s*_min_ ≤ *s* ≤ *s*_max_. The curves for each range of *q* or *s* used in the fitting are shifted vertically for visualization purposes.

**Figure 6:**
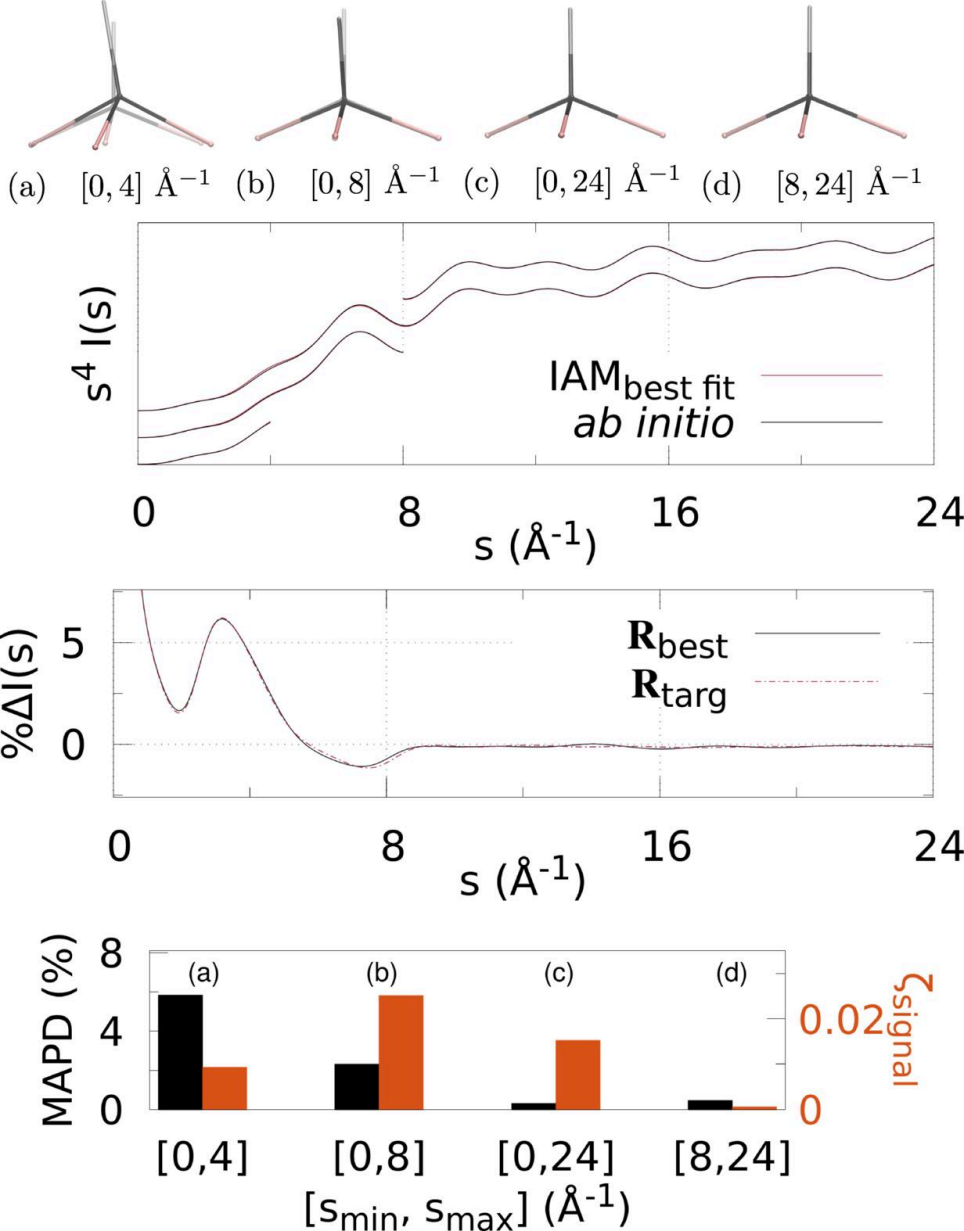
Electron scattering of CHF_3_ using different *s*-ranges for structure determination: (*a*)–(*d*) The best fit geometry for each *s*-range (solid) versus the reference structure **R**_targ_ (translucent), where *s* ∈ [*s*_min_, *s*_max_] = [0, 4], [0, 8], [0, 24], [8, 24] Å^−1^. (Top) The IAM best fits to the *ab initio* electron scattering calculated at **R**_targ_ as a function of *s*-range. The *s*_max_ > 4 Å^−1^ curves are shifted vertically for visibility. (Middle) The percent difference 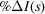 [equation (3)] (solid black line) for the lowest ζ_targ_ structure. For comparison, 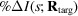 is also shown, using *I*_IAM_(*s*; **R**_targ_) and *I*_abinitio_(*s*; **R**_targ_). (Bottom) Bar chart showing the MAPD [equation (6)] and ζ_signal_ for each value of [*s*_min_, *s*_max_].

The bar charts at the bottom summarize the fitting for each signal range in terms of ζ_signal_ (notably, not ζ_targ_) as in equation (4), and MAPD [equation (6)]. Tables 1 ▸ and 2 ▸ show the values from these charts. It is clear that 8 ≤ *q* ≤ 12 Å^−1^ finds the molecular geometry closest to **R**_targ_; in fact, it is exceptionally close to the target, with mean absolute atom–atom distance deviations 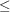 0.9% for all three molecules. Similarly, for electron scattering, the range 8 ≤ *s* ≤ 24 Å^−1^ gives the best geometry with MAPD 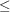 0.5%. This can be seen clearly from the overlap of the solid and translucent structures in (*e*) [or (*d*) for Figs. 6 ▸–8 ▸ ▸], whereas (*a*), (*b*), *etc*. have quite large visible deviations from **R**_targ_, such as stretching of C—F bonds, aromatic C—C bonds compacted/stretched, and different hydrogen positions (and C—H distances). Importantly, MAPD and ζ_signal_ are generally correlated (aside from the smallest electron scattering range 0 ≤ *s* ≤ 4 Å^−1^ outliers), and the high-*q* (or *s*) ranges 8 ≤ *q* ≤ 12 Å^−1^ and 8 ≤ *s* ≤ 24 Å^−1^ have the lowest MAPD and ζ_signal_ in all cases except for one outlier in the CHF_3_ electron scattering where the 0 ≤ *s* ≤ 24 Å^−1^ range has slightly lower (albeit very close) MAPD, despite much higher ζ_signal_. The correlation between MAPD and ζ_signal_ is still promising however, as experimentally we do not know the MAPD (because we do not inherently know the molecular geometry) but do know the value of ζ_signal_ from comparison with theory. Therefore, fitting the X-ray (or electron) scattering curve via minimization of ζ_signal_ (or equivalent) should give a structure close to the true structure.

Finally, the middle graphs shows the valence electronic structure component as a percentage, 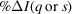 , for the best-fit geometry, **R**_best_, *i.e.* the structure with lowest ζ_signal_ for the ranges 8 ≤ *q* ≤ 12 Å^−1^ and 8 ≤ *s* ≤ 24 Å^−1^ for X-ray and electron scattering, respectively, using the *ab initio* signal calculated at **R**_targ_ as the reference. It is compared with the percent differences between IAM and *ab initio* both at **R**_targ_ (dashed red line). The results show good agreement between 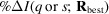 and 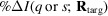 for both X-ray and electron scattering. Notably, the electron results find near perfect agreement in this regard, revealing that **R**_best_ is closer to **R**_targ_ compared with the X-ray data fitting, hinting that electron scattering could be a better tool for molecular structure determination at larger scattering vector amplitudes compared with X-ray scattering. This is likely due to the dominance of elastic electron scattering at high values of *s*, whereas high-*q* X-ray scattering is dominated by unmodulated inelastic scattering. Despite this, X-ray scattering still performed well in this region, finding low MAPD structures for each molecule. It is striking that the elastic scattering structural information persists here (see Fig. S2 of the supporting information).

## 4. Conclusion

The molecular geometry can be determined from large momentum transfer or equivalently large-angle scattering, with *q* > 8 Å^−1^ (in the following, *q* also encompasses *s*), with good results achieved already with the simple IAM approximation if only the large-*q* data are used. Although the elastic scattering component containing structural information drops off more quickly for X-ray than electron scattering, we find that sufficient elastic scattering persists to retrieve the correct molecular geometry using IAM theory in the range 8 < *q* < 12 Å^−1^ for both modalities of scattering. An important aspect to note is that in this high *q* regime the contribution to the scattering due to chemical bonding is negligible, allowing the structure to be determined reliably using IAM theory. Conversely, if using IAM across the full available range of *q*, the resulting structure may be distorted from the correct **R**_0_ geometry. Another point to note is that the concept of structure may become ill-defined in excited-state dynamics, where the dispersion of the nuclear wave packet leads to the co­existence of a range of structures that manifest as an effective damping of the high-*q* signal (Kirrander & Weber, 2017 ▸).

Nevertheless, for states of reasonably well defined geometry, such as molecules in their ground electronic state, we can determine the molecular geometry using the large-*q* scattering, allowing us in the next step to extract the contribution to the scattering from the bonding valence electrons, at small and intermediate *q*. In this *q*-range, we find the deviation from IAM to be significant: ∼10% for CHD and naphthalene, and ∼5% for CHF_3_, for both X-ray and electron scattering.

We note that any robust inversion algorithm that can transform the one-dimensional scattering signal into reasonable molecular geometries would work (Yang *et al.*, 2014 ▸; Ishikawa *et al.*, 2015 ▸; Acheson & Kirrander, 2023 ▸). A practical challenge is that the large-*q* signal is small and is detected on a background of featureless inelastic scattering, which is demanding in terms of experimental signal-to-noise. The reliable detection of large-*q* signals might therefore be most appropriate at facilities such as the upgraded LCLS-II where high repetition rates and photon energies upwards of 18 keV can help overcome such shortcomings. Nevertheless, given data of sufficient quality, the procedure outlined in this paper demonstrates that it should be possible to isolate the electronic contributions to the scattering signal, potentially opening the door for exciting new insights into electronic structure (Carrascosa *et al.*, 2022 ▸).

## 5. Related literature

The following reference, not cited in the main body of the paper, has been cited in the supporting information: Mai *et al.* (2014 ▸).

## Figures and Tables

**Figure 4:**
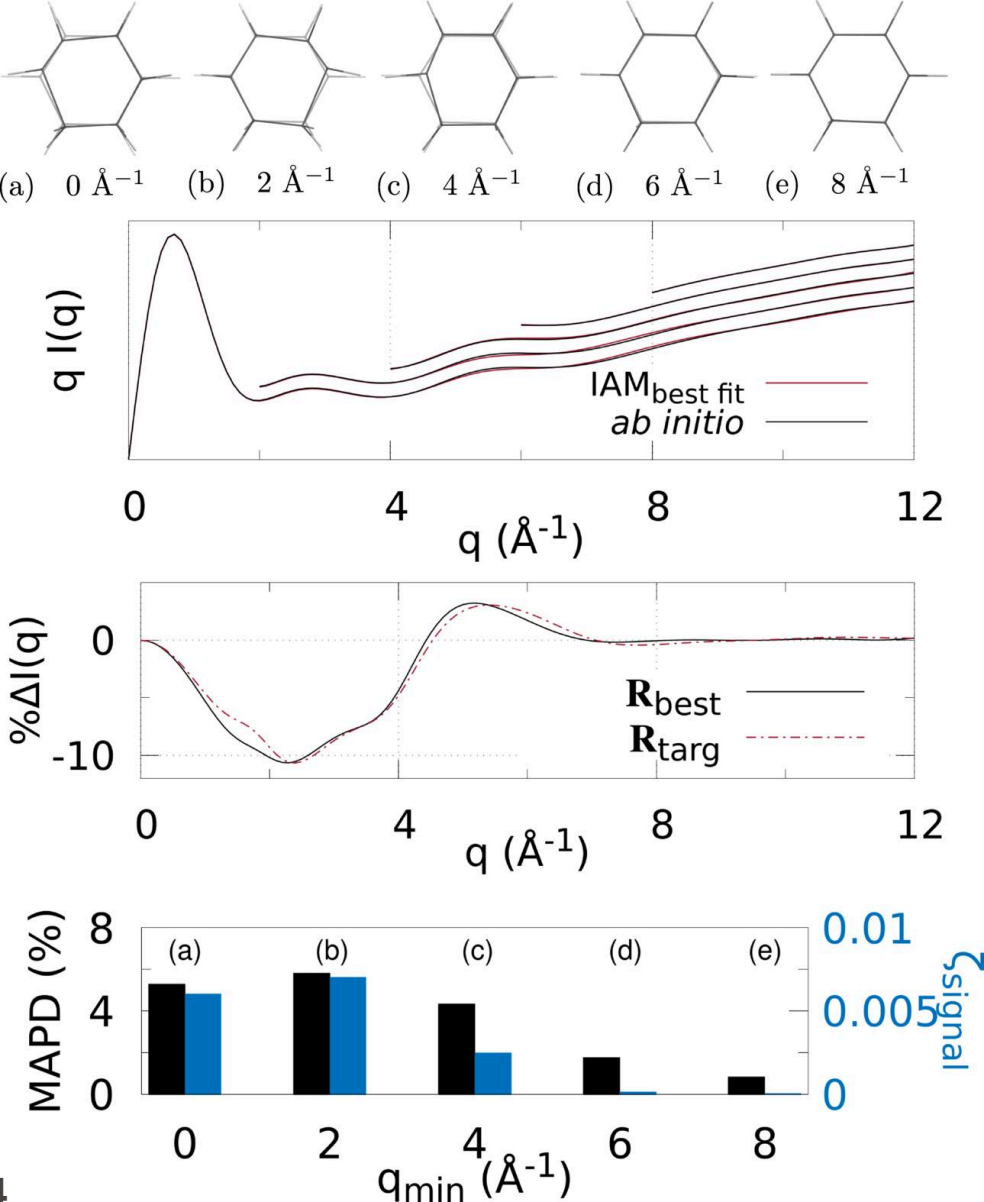
X-ray scattering of CHD using different *q*-ranges for structure determination: (*a*)–(*e*) The best fit geometry for each *q*_min_ (solid) versus the target structure **R**_targ_ (translucent), where *q* ∈ [*q*_min_, *q*_max_], and *q*_min_ = [0, 2, 4, 6, 8] Å^−1^ and *q*_max_ = 12 Å^−1^. (Top) The IAM best fits to the *ab initio* X-ray scattering calculated at **R**_targ_ as a function of *q*-range. The *q*_min_ ≥ 2 Å^−1^ curves are shifted vertically for visibility. (Middle) The percent difference 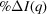 [equation (3)] (solid black line) for the lowest ζ_targ_ structure. For comparison, 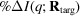 is also shown, using *I*_IAM_(*q*; **R**_targ_) and *I*_abinitio_(*q*; **R**_targ_). (Bottom) Bar chart showing the MAPD [equation (6)] and ζ_signal_ for each value of *q*_min_.

**Figure 5:**
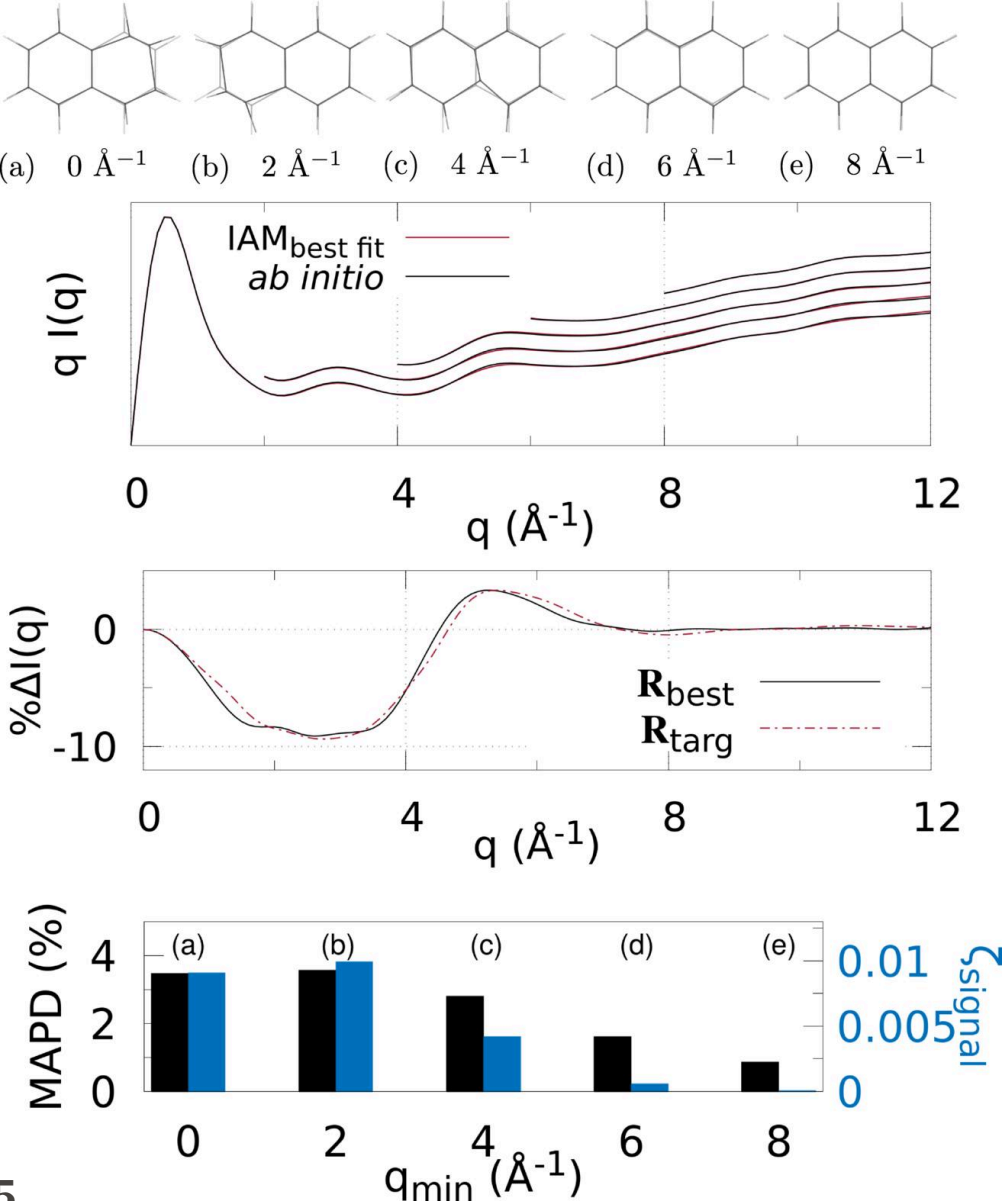
X-ray scattering of naphthalene using different *q*-ranges for structure determination: (*a*)–(*e*) The best fit geometry for each *q*_min_ (solid) versus the target structure **R**_targ_ (translucent), where *q* ∈ [*q*_min_, *q*_max_], and *q*_min_ = [0, 2, 4, 6, 8] Å^−1^ and *q*_max_ = 12 Å^−1^. (Top) The IAM best fits to the *ab initio* X-ray scattering calculated at **R**_targ_ as a function of *q*-range. The *q*_min_ ≥ 2 Å^−1^ curves are shifted vertically for visibility. (Middle) The percent difference 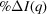 [equation (3)] (solid black line) for the lowest ζ_targ_ structure. For comparison, 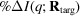 is also shown, using *I*_IAM_(*q*; **R**_targ_) and *I*_abinitio_(*q*; **R**_targ_). (Bottom) Bar chart showing the MAPD [equation (6)] and ζ_signal_ for each value of *q*_min_.

**Figure 7:**
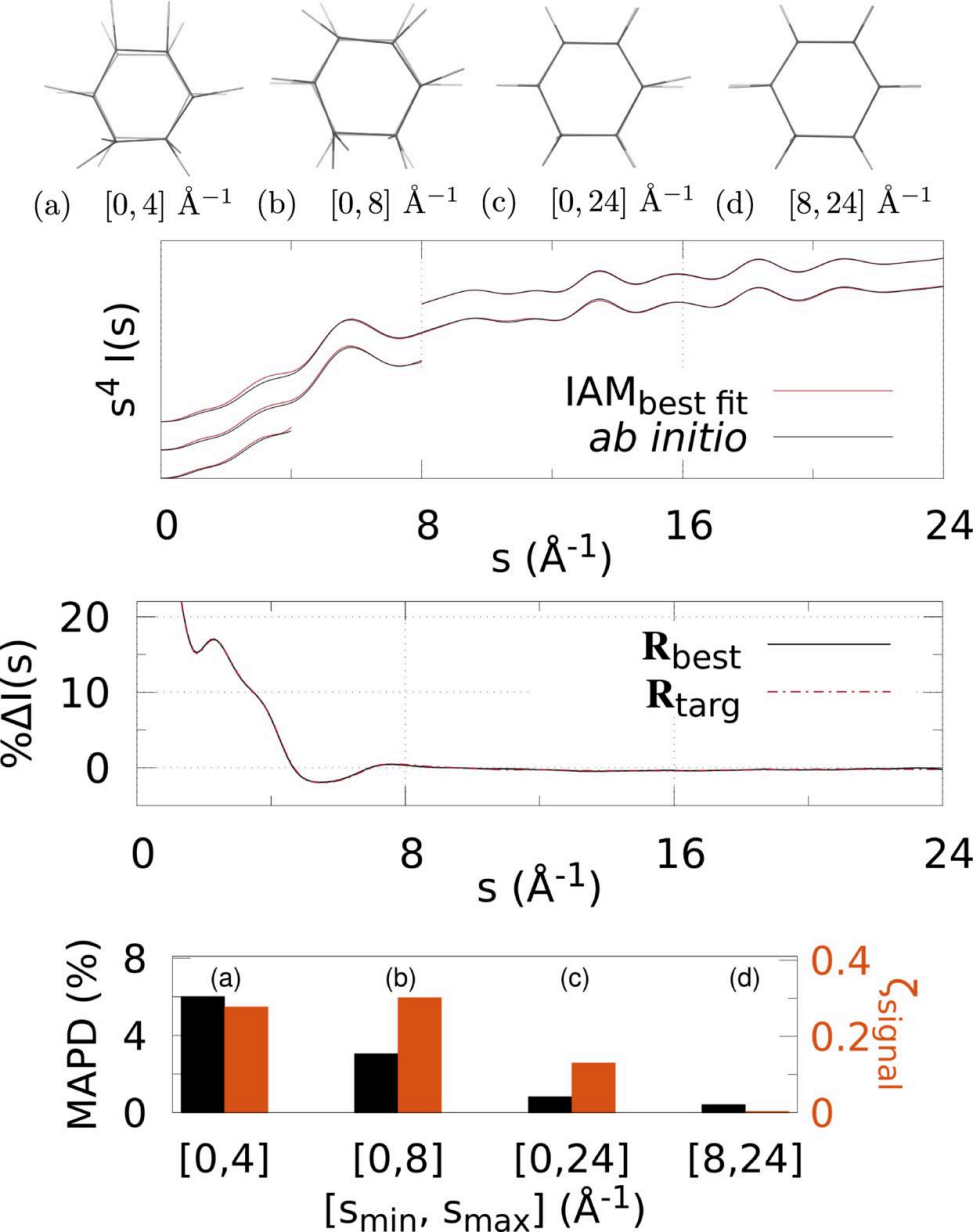
Electron scattering of CHD using different *s*-ranges for structure determination: (*a*)–(*d*) The best fit geometry for each *s*-range (solid) versus the reference structure **R**_targ_ (translucent), where *s* ∈ [*s*_min_, *s*_max_] = [0, 4], [0, 8], [0, 24], [8, 24] Å^−1^. (Top) The IAM best fits to the *ab initio* electron scattering calculated at **R**_targ_ as a function of *s*-range. The *s*_max_ > 4 Å^−1^ curves are shifted vertically for visibility. (Middle) The percent difference 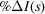 [equation (3)] (solid black line) for the lowest ζ_targ_ structure. For comparison, 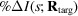 is also shown, using *I*_IAM_(*s*; **R**_targ_) and *I*_abinitio_(*s*; **R**_targ_). (Bottom) Bar chart showing the MAPD [equation (6)] and ζ_signal_ for each value of [*s*_min_, *s*_max_].

**Figure 8:**
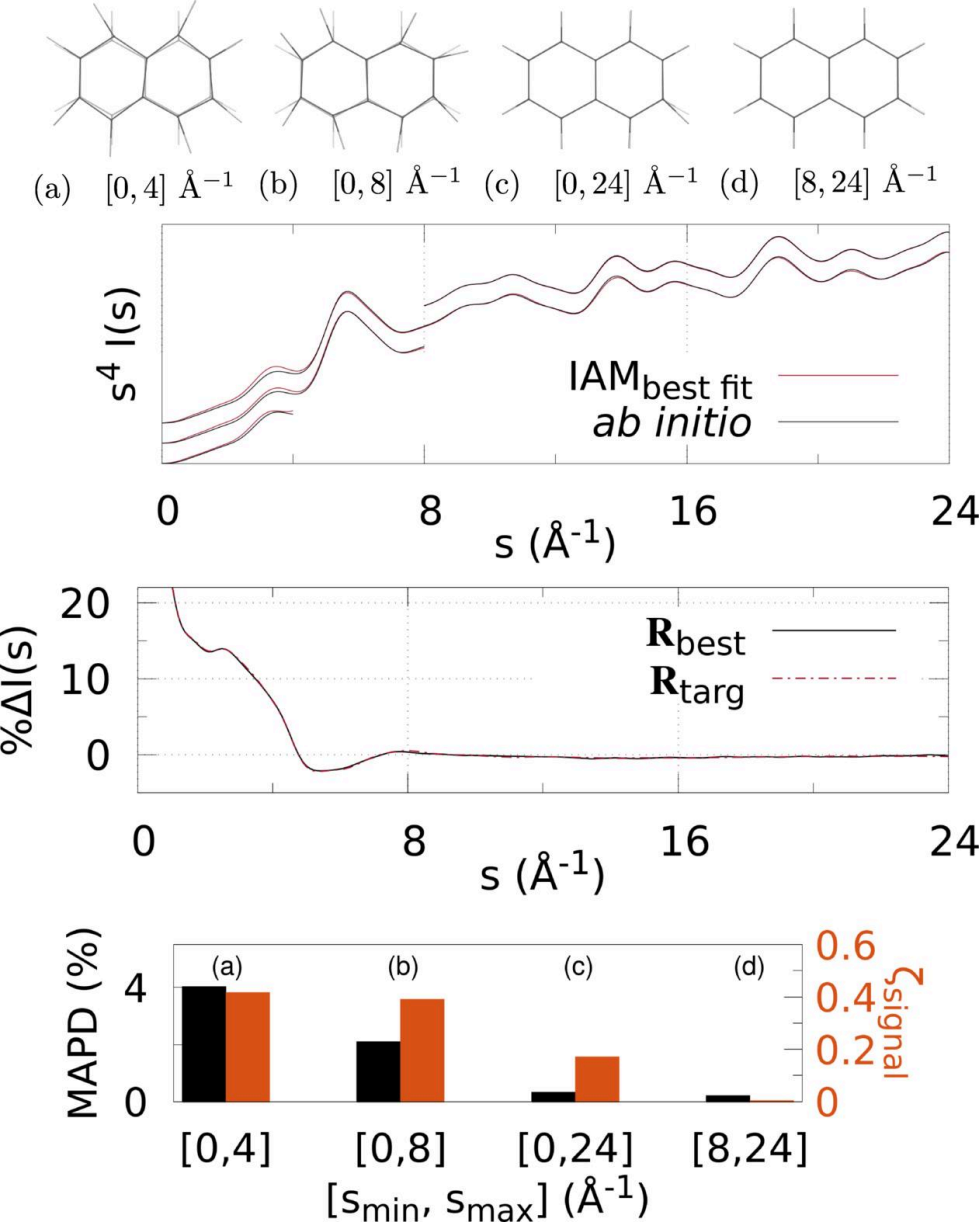
Electron scattering of naphthalene using different *s*-ranges for structure determination: (*a*)–(*d*) The best fit geometry for each *s*-range (solid) versus the reference structure **R**_targ_ (translucent), where *s* ∈ [*s*_min_, *s*_max_] = [0, 4], [0, 8], [0, 24], [8, 24] Å^−1^. (Top) The IAM best fits to the *ab initio* electron scattering calculated at **R**_targ_ as a function of *s*-range. The *s*_max_ > 4 Å^−1^ curves are shifted vertically for visibility. (Middle) The percent difference 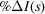 [equation (3)] (solid black line) for the lowest ζ_targ_ structure. For comparison, 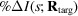 is also shown, using *I*_IAM_(*s*; **R**_targ_) and *I*_abinitio_(*s*; **R**_targ_). (Bottom) Bar chart showing the MAPD [equation (6)] and ζ_signal_ for each value of [*s*_min_, *s*_max_].

**Table 1:** Fitting results between IAM X-ray scattering and *ab initio* calculated at the target geometry **R**_targ_ at different *q*-ranges *q*_min_ is shown in the first column and *q*_max_ = 12 Å^−1^, and the data correspond to the bar charts in Figs. 3 ▸–5 ▸ ▸. The target function, ζ_signal_, is described in equation (4), and the mean absolute percent deviation (MAPD) is given by equation (6)

*q*_min_ (Å^−1^)	ζ_signal_	MAPD (%)
CHF_3_		
0	1.0 × 10^−2^	4.5
2	9.5 × 10^−3^	4.2
4	2.7 × 10^−3^	1.3
6	1.9 × 10^−3^	1.9
8	4.7 × 10^−4^	0.2

CHD		
0	6.0 × 10^−3^	5.3
2	7.0 × 10^−3^	5.8
4	2.5 × 10^−3^	4.3
6	1.3 × 10^−4^	1.7
8	1.6 × 10^−5^	0.8

Naphthalene		
0	9.1 × 10^−3^	3.5
2	9.9 × 10^−3^	3.6
4	4.2 × 10^−3^	2.8
6	5.8 × 10^−4^	1.6
8	2.4 × 10^−5^	0.9

**Table 2:** Fitting results between IAM electron scattering and *ab initio* calculated at the target geometry **R**_targ_ at different *s*-ranges *s*_min_ is shown in the first column and *s*_max_ in the second, and the data correspond to the bar charts in Figs. 6 ▸ ▸–8 ▸. The target function, ζ_signal_, is described in equation (4), and the mean absolute percent deviation (MAPD) is given by equation (6).

*s*_min_ (Å^−1^)	*s*_max_ (Å^−1^)	ζ_signal_	MAPD (%)
CHF_3_			
0	4	9.3 × 10^−3^	5.8
0	8	2.5 × 10^−2^	2.3
0	24	1.5 × 10^−2^	0.3
8	24	5.1 × 10^−4^	0.5

CHD			
0	4	2.8 × 10^−1^	6.0
0	8	3.0 × 10^−1^	3.0
0	24	1.3 × 10^−1^	0.8
8	24	1.9 × 10^−3^	0.4

Naphthalene			
0	4	4.2 × 10^−1^	4.0
0	8	3.9 × 10^−1^	2.1
0	24	1.7 × 10^−1^	0.3
8	24	3.2 × 10^−3^	0.2
